# Value drivers of development stage biopharma companies

**DOI:** 10.1007/s10198-021-01427-5

**Published:** 2022-01-17

**Authors:** Daniel Tobias Michaeli, Hasan Basri Yagmur, Timur Achmadeev, Thomas Michaeli

**Affiliations:** 1 Fifth Department of Medicine, University Hospital Mannheim, Heidelberg University, Mannheim, Germany; 2 Division of Personalized Oncology, University Hospital Mannheim, Heidelberg University, Mannheim, Germany; 3 TUM School of Management, Technical University of Munich, Munich, Germany; 4 Division of Personalized Medical Oncology, German Cancer Research Center (DKFZ), Heidelberg, Germany

**Keywords:** Multi-indication, Pharmaceutical policy, Orphan, Valuation, Acquisitions, Drug development

## Abstract

**Objective:**

Scholars previously estimated research and development (R&D) costs of the internal drug development process. However, little is known about the costs and value arising from externally acquired therapeutics. This study identifies and estimates the magnitude of factors associated with Biopharma acquisition value.

**Methods:**

SDC Thomson Reuter and S&P Capital IQ were screened for majority acquisitions of US and EU Biopharma companies developing new molecular entities for prescription use (SIC code: 2834) from 2005 to 2020. Financial acquisition data were complemented with variables characterizing the target’s product portfolio extracted from clinicaltrials.gov, Drugs@FDA database, US SEC filings, and transaction announcements. A multivariate regression assesses the association of firm value with extracted variables.

**Results:**

311 acquisitions of companies developing prescription drugs were identified over the study period. Acquirers paid 37% (*p* < 0.05) more for companies with biologics and gene therapeutics than small-molecule lead drugs. Multi-indication products were acquired for a 12% premium per additional indication (*p* < 0.01). No significant valuation difference between companies developing orphan and non-orphan designated lead products was observed (18%, *p* = 0.223). Acquisition value positively correlated with the total number of further products, headquarter location in the US, underlying market conditions, and acquirer market capitalization (*p* < 0.05).

**Conclusions:**

Internal and external drug development consumes many financial and human resources, yet it is important for entrepreneurs, regulators, and payers to understand their precise magnitude and value drivers. This information permits the design of targeted pricing and industrial policies that incentivize the development of novel drugs in areas with high unmet needs.

**Supplementary Information:**

The online version contains supplementary material available at 10.1007/s10198-021-01427-5.

## Introduction

Rising drug prices recently sparked controversy about high profit margins of pharmaceutical companies [1, 2]. Crucial to this dispute are the costs associated with developing new drugs [3]. While scholars previously estimated research and development (R&D) costs of the internal drug development process [4–6], little is known about the value and costs associated with externally developed therapeutics [7].

Estimates show that the share of revenue from novel drugs developed externally surged to 50% in 2016 [7]. External innovation sources include partnerships with academic institutions, licensing agreements, and mergers and acquisitions (M&A) of disruptive start-ups. Acquisitions may be especially advantageous for strategic Biopharma companies when internal R&D pipelines must be replenished quickly due to patent expiry [8, 9]. Furthermore, partnerships and acquisitions combine leading technological advances from risk-tolerant incumbent biotechnology start-ups with established commercialisation capabilities of large pharmaceutical companies [10]. These synergies do not only create direct value for acquired start-ups, venture capital investors, and large pharmaceutical corporations, but could ultimately benefit patients by permitting a timely access to innovative medicines. Concisely, acquisitions fuel the development of medicines with financial, human, and technological capital which eventually advances available therapeutic options.

Innovation combined with a high risk-return profile has long sparked the interest of venture capitalists in the Biopharma industry. After several funding stages successful start-ups either debut on a public stock exchange through an initial public offering (IPO) or are sold directly to strategic or financial investors. Despite available economic valuation methodologies, e.g., net present value (NPV), risk-adjusted NPV (rNPV), real options, or the venture capital method, the valuation of Biopharma companies remains challenging due to the absence of solid financial metrics [11–13]. Even though there are some attempts to account for the intangible value of pharmaceutical companies arising from technological firm capabilities or patents [14, 15], such approaches are still imperfect. Greater knowledge of external Biopharma innovation sources can inform the design of pricing and industrial policies that effectively reward the development of novel drugs in areas with high unmet needs [16–19].

Evidently, Biopharma firm valuation is mainly subject to the lead product’s development stage [10, 20–23]. However, knowledge on factors that explain the valuation dispersion within development stages is scarce. Yearly Biopharma deal reviews often focus on multi-billion-dollar acquisitions [10, 21]. Thereby, early-stage pre-clinical and clinical stage acquisitions, which drive pharmaceutical innovation, are neglected. A regression analysis of 122 US Biopharma IPOs (1991–2000) found significant correlations between firm value and the products’ development stage, R&D expenditure, market conditions, ownership retention, as well as a company’s number of total products, alliances, and patents [22]. A cross-sectional study of 98 M&As (2008–2012) revealed no significant valuation difference between companies with US Food and Drug Administration (FDA) orphan and non-orphan designated lead products [24]. Valuations were also identified to be higher for US, large cap pharma-backed, and oncology companies [25, 26]. Surveys with 16 financial and strategic investors in 2002 qualitatively identified market size, development stage, strategic fit to acquirer, competition, reputation, patents, and product novelty—in this order—as most important value drivers in Biopharma licensing deals [23].

Previous studies are, therefore, limited in sample size, geographic scope, and breadth of examined variables. Our study fills this gap by quantitatively assessing Biopharma company valuations based on a sample of 311 M&As across 23 collected variables in the US and EU between 2005 and 2020. We specifically aim to examine the correlation between company acquisition value and the lead product’s development stage (Pre-Clinic to FDA Approval), additional lead product, other products, and transition variables using multivariate regression analyses. To the best of our knowledge, this is the first study that identifies and quantifies key financial and non-financial value drivers of private and public Biopharma corporations.

## Data and methods

### Sample selection

SDC Thomson Reuter and S&P Capital IQ were screened for majority acquisitions of Biopharma companies developing new molecular entities (NME) for therapeutic use (SIC code: 2834) from 01.01.2005 to 01.01.2020. Corporations developing generics, reformulations, medical devices, diagnostic substances, over-the-counter medicines, cannabis products, animal therapeutics as well as active pharmaceutical ingredients producers and sales of manufacturing sites were excluded. Only acquisitions with a total transaction value beyond $10 million were considered. To exclude mega mergers, the sample was limited to targets with a portfolio of less than 10 NME. The geographic location was restricted to targets headquartered in the US or developed European markets. The sample contains both private and public targets.

### Data collection

Variables were collected across four distinct areas: valuation, lead product, further products, and acquisition characteristics (Table 1). Selection was based on previous quantitative and qualitative studies that identified variables associated with Biopharma firm value [22–26]. Financial variables and acquisition characteristics were extracted from SDC Thomson Reuter and S&P Capital IQ. Subsequently, variables characterizing the target’s product portfolio were obtained from US Securities and Exchange Commission (SEC) filings, clinicaltrials.gov, transaction announcements, and company websites at the time of acquisition announcement.

**Table 1:** Descriptive statistics for the entire sample

	Unit	*N*	Mean	Median	SD	Min	Max	Skewness	Sum
*(A) Valuation*									
Up-front payment	USD	303	958	257	2270	0	22,434	5.02	290,417
Milestone payment	USD	290	159	0	330	0	2945	3.89	46,144
Total transaction value	USD	300	1119	458	2285	12	22,434	4.86	335,662
*(B) Lead drug*									
Development stage									
Pre-Clinic	Binary	311	0.15	0.00	0.36	0	1		46
Phase 1	Binary	311	0.13	0.00	0.34	0	1		40
Phase 2	Binary	311	0.33	0.00	0.47	0	1		103
Phase 3	Binary	311	0.19	0.00	0.39	0	1		59
Approved	Binary	311	0.20	0.00	0.40	0	1		63
No. of indications	Number	311	1.83	1.00	1.88	1	16	4.20	569
Biologic/gene therapy	Binary	311	0.21	0.00	0.41	0	1		66
Disease area									
Oncology	Binary	311	0.30	0.00	0.46	0	1		93
CNS	Binary	311	0.16	0.00	0.37	0	1		51
Anti-viral/anti-biotic	Binary	311	0.11	0.00	0.32	0	1		35
Others	Binary	311	0.42	0.00	0.50	0	1		132
FDA orphan designation	Binary	311	0.16	0.00	0.37	0	1		51
*(C) Other products*									
Total no. of drugs	Number	311	2.96	2.00	2.19	1	10	1.31	921
Average development score^a^	Number	311	6.38	5.67	3.67	2	14	0.76	1984
Average no. of indications^b^	Number	311	1.46	1.00	1.09	1	12	5.00	455
*(D) Acquisition characteristics*									
Target headquarter US	Binary	311	0.76	1.00	0.43	0	1		235
Target public ownership	Binary	311	0.37	0.00	0.48	0	1		114
Acquirer market cap ≥ $10 Bn	Binary	311	0.47	0.00	0.50	0	1		147
Market conditions	Number	311	0.12	0.10	0.21	− 0.24	0.69	0.47	38
Spin-off/single drug acquisition	Binary	311	0.11	0.00	0.31	0	1		34

All valuation metrics are inflation adjusted

*SD* standard deviation, *CNS* central nervous system, *FDA* US Food and Drug Administration, *HQ* headquarter

^a^The average development score represents the number of years required to reach each development stage

^b^The average number of indications refers to all products excluding the lead product

#### Valuation metrics

Up-front payments, maximum milestone payments (both regulatory and sales), and the total transaction value were obtained from SDC Thomson Reuter and S&P Capital IQ in US dollars at the time of the acquisition. To ensure data validity, all company valuations were cross-checked with US SEC filings and transaction announcements, if available. Valuation metrics were adjusted for inflation to 2020 values.

#### Lead product characteristics

We obtained multiple variables characterizing the target’s lead product. For clinical phase products, the development stage was extracted from clinicaltrials.gov. Therapeutics in parallel Phase 1/2 trials were categorized within the Phase 2 development stage. For approved products, the development stage was derived from public available marketing authorization reports issued by the FDA. For pre-clinical products, the development stage was derived from US SEC filings or transaction announcements. The same methodology was applied to identify and categorize the lead product’s number of indications (single indication vs. multi-indication), treatment type (small-molecule vs. biologic/gene and cell therapy), and disease area (oncology, central nervous system (CNS), infectious diseases, and others) according to the most advanced indication.

#### Further products

The same methodology was employed to obtain the target’s total number of medicines alongside their development stage, and number of indications. We applied a similar concept proposed by Guo et al. to calculate the remaining portfolio’s average development stage [22, 27]. The stage score represents the number of years required to reach each development stage. Consistently, the average number of indications of all further products was assessed.

#### Acquisition characteristics

Target ownership status (private vs. public) and headquarter location (Europe vs. US) was extracted. We further identified the asset type (company acquisitions vs. spin-off/single drug transactions). The market condition variable represents the dividend and stock-split adjusted return of the NASDAQ Biotech—an index capturing the market capitalization of NASDAQ listed Biopharma companies according to the SIC code—12 months prior to transaction announcement.

### Methods and statistical analysis

Data were stored in Microsoft EXCEL and then analyzed using STATA SE Version 15.1. We calculated mean acquisition values and payment structures across our sample. Data were expressed as means with 95% confidence intervals (CI). Company valuations were compared across development stages using ANOVA with Turkey’s multiple comparison test. A two-tailed probability value < 0.05 was considered significant.

Thereafter, valuation metrics were examined in a sequence of multivariate regression models. First, valuation metrics were transformed with the natural logarithm to account for the right skewed data distribution. Several regressions are presented in a consistent stepwise structure to examine the association of collected variables with company valuation. Model 1 only includes the lead product’s development stage as explanatory variable. Model 2 further includes all lead product characteristics. Model 3 considers all lead product and further product variables. Model 4 entails all lead product, further product, and acquisition variables. Model 5 presents an optimized regression that excludes multicollinearity among explanatory variables (Supplementary Tables e1 and e2). This sequence of regression models permits to assess the explanatory value, measured by Adjusted-R^2^, of the different variable categories. Mathematical equations for all regression models are attached in Supplementary Box e1. Post-regression tests were conducted, as shown in Supplementary Table e3, to evaluate omitted variable bias (Ramsey’s test), model specification errors (Link test), as well as heteroscedasticity, skewness, and kurtosis (Cameron and Trivedi’s test).

## Results

**Fig. 1:**
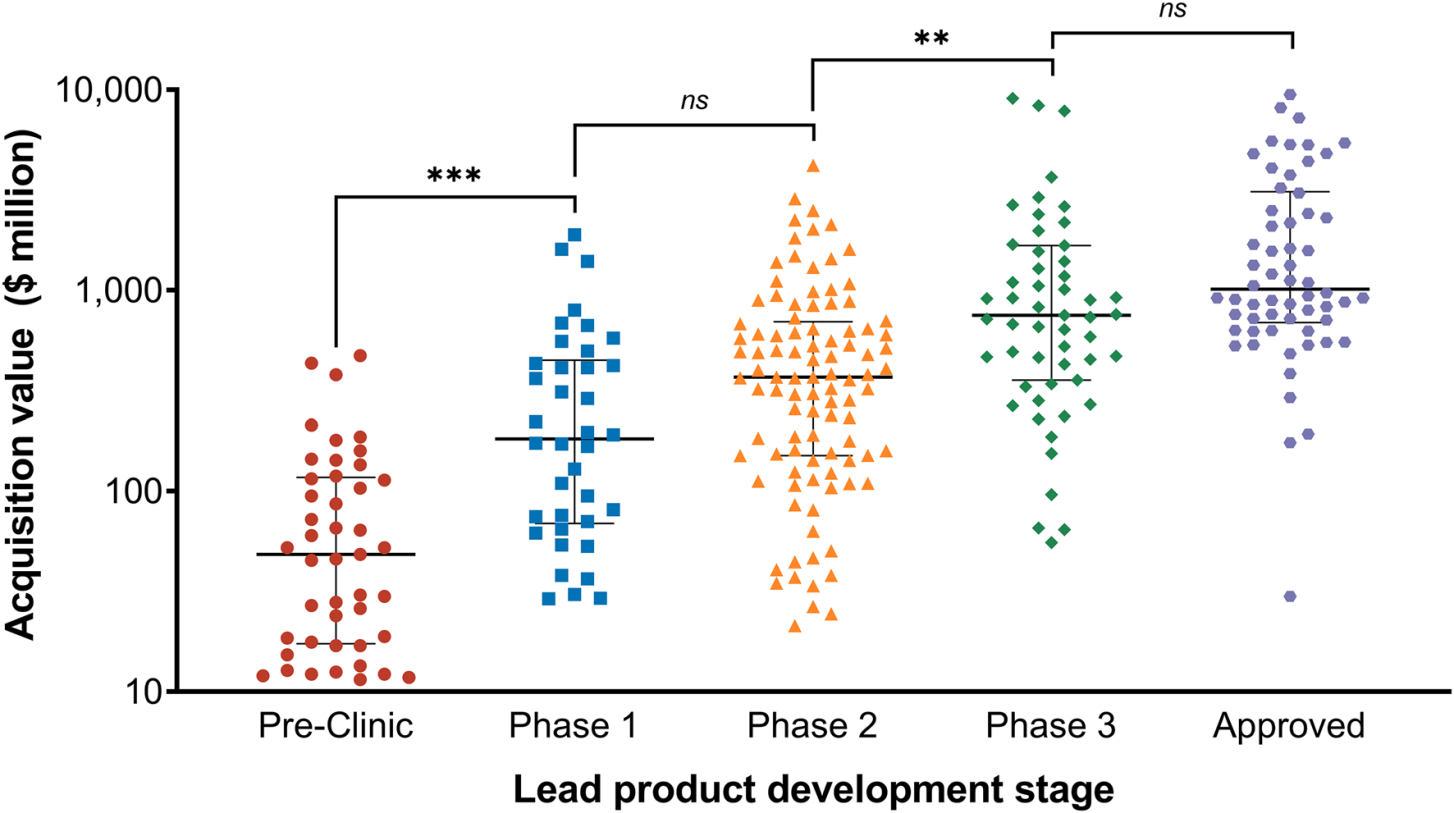
Acquisition value of Biopharma companies by lead product development stage. All values were inflation adjusted to 2020. *p* values calculated based ANOVA with Turkey’s multiple comparison test: **p < 0.01, *** *p* < 0.001, ns not significant

Overall, we identified 2106 unique Biopharma acquisitions in the SDC Thomson Reuter (*n* = 1427) and S&P Capital IQ (*n* = 679) databases between 01.01.2005 and 01.01.2020 with valuation metrics (Fig. 1). Further restricting the search to companies developing NME for human prescription use led to a final sample of 311 Biopharma M&As.

### Descriptive statistics

**Fig. 2:**
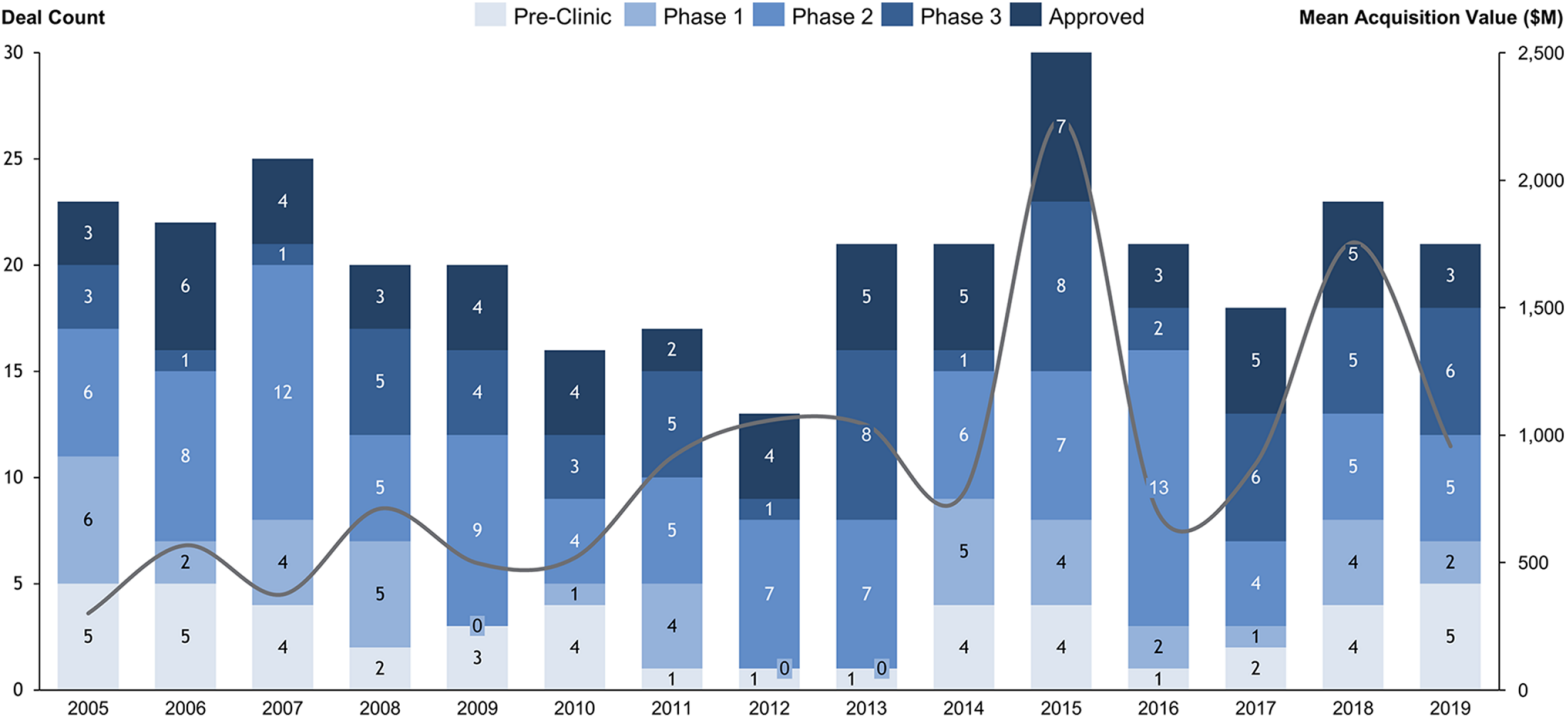
Number and deal value of development stage Biopharma acquisitions from 2005 to 2020. The deal count represents the number of acquisitions by development stage. Mean acquisition values are inflation adjusted to 2020

Overall, the entire acquisition volume cumulated to $336 billion over the 15-year period (Fig. 2). On average, firms were acquired for a total transaction value of $1119 million (up-front payment: $958 million; milestone payment: $159 million). Valuation metrics were not reported for the entire sample (total transaction value: 300; up-front payment: 303; milestone payment: 290).

Most acquired corporations were developing a lead product in Phase 2 (33%) or already commercialized the lead product (20%). Approximately one third of lead products were developed across multiple indications (Table 1). 21% were classified as biologics or gene therapy and 16% received an orphan designation from the FDA. Most acquisitions focused on oncology (30%), CNS (16%), and infectious diseases therapies (11%).

On average, acquired companies had a product portfolio of approximately 3 medicines. Yet, one third of companies only pursued the development of one product. The average development score of these further products was 4.18, indicating that the average portfolio of the further products was between the Pre-Clinical and Phase 1 development stage. Only 18% of further products under development were tested across several indications.

Target companies were mostly headquartered in the US (76%) and under private ownership (37%). Furthermore, 47% of corporations were acquired by companies with a total market capitalization of more than $10 billion. The sample also includes acquisitions of single drugs or spin-offs (11%). Acquisitions were predominantly struck under favorable market conditions, with the NASDAQ Biotech index posting an average 12-month return of approximately 12% prior to transaction announcement.

#### Valuation by development stage

On average, companies with lead products in pre-clinical development were acquired for a total transaction value of $88 million (95% CI $56–120 million). Mean valuations rose to $354 million (95% CI $211–498 million) for Phase 1, $683 million (95% CI $436–930 million) for Phase 2, $1761 million (95% CI $996–2527 million) for Phase 3, and $2469 million (95% CI $1582–3355 million) for Approved lead products. However, the acquisition value displayed a high dispersion within development stages. Therefore, only the transitions from Pre-Clinic to Phase 1 (*p* < 0.001) and from Phase 2 to Phase 3 (*p* < 0.01) significantly differed.

#### Payment structure

Acquisitions of companies with lead products under Pre-clinic and Phase 1 development included a mean up-front component of 51% (95% CI 49–75%) and 43% (95% CI 51–74%), respectively. Consequently, approximately half of early-stage company transaction value was a deferred component (milestone payment). However, this deferred component decreased while the up-front component increased throughout clinical development. The mean up-front payment amounted to 72% (95% CI 56–71%) for Phase 2, 80% (95% CI 69–86%) for Phase 3, and 95% (95% CI 85–95%) for approved lead products.

### Multivariate regression

The association between total transaction value and collected variables is evaluated in a sequence of multivariate regression models. Lead product, further product, and transaction characteristics are separately examined in a sequence of stepwise regressions (Table 2).

**Table 2:** Multivariate regression of total transaction value on (A) lead product’s, (B) other products’, and (C) acquisition characteristics

	Model 1	Model 2	Model 3	Model 4	Model 5
*Dependent variable: natural logarithm of total transaction value*					
(A) Lead product					
Phase 1	1.338***	1.281***	1.102***	0.861***	0.967***
Phase 2	1.914***	1.928***	1.652***	1.302***	1.492***
Phase 3	2.788***	2.776***	2.483***	1.918***	2.277***
Approved	3.302***	3.092***	2.637***	1.987***	2.532***
No. of indications		0.128***	0.131	0.080	0.119**
Biologic/gene therapy		0.474**	0.452**	0.340*	0.374*
Oncology		0.110	0.085	0.058	0.057
CNS		− 0.270	− 0.284	− 0.306	− 0.290
Anti-viral/anti-biotic		0.092	0.120	0.058	0.058
FDA orphan designation		0.192	0.098	0.172	0.180
(B) Other products					
Total no. of drugs			0.184***	0.174***	0.152***
Average development score^a^			0.028	0.043	
Average no. of indications^b^			− 0.016	0.106	
(C) Acquisition characteristics					
Target headquarter US				0.593***	0.634***
Target public ownership				0.189	
Acquirer market cap ≥ $10 Bn				0.761***	0.732***
Market conditions				0.780**	0.791**
Spin-off/single drug acquisition				− 0.404*	− 0.343
Constant	3.890***	3.579***	3.153***	2.493***	2.693***
No. of observations	300	300	300	300	300
*R*^2^	44.9%	50.2%	55.5%	66.7%	66.2%
Adjusted-*R*^2^	44.1%	48.5%	53.4%	64.5%	64.4%
*F *test					
Pre-Clinic to Phase 1	1.338***	1.281***	1.102***	0.861***	0.967***
Phase 1 to 2	0.576*	0.647**	0.550*	0.441*	0.525**
Phase 2 to 3	0.875***	0.848***	0.831***	0.616**	0.785***
Phase 3 to Approved	0.514*	0.316	0.154	0.069	0.255

*CNS* central nervous system, *FDA* US Food and Drug Administration, *HQ* headquarter

*p* values: *p < 0.05, **p < 0.01, ***p < 0.001, *NS* not significant

^a^The average development score represents the number of years required to reach each development stage

^b^The average number of indications refers to all products excluding the lead product

#### Lead product characteristics

The lead product’s development stage is the major highly significant value driver, explaining approximately 44.1% of firm valuation (Model 1). The total transaction value significantly increased with the lead product’s number of indications (*p* < 0.001). Companies developing biologics or gene therapeutics were sold for a 37.4% (*p* < 0.05) premium relative to small-molecule medicines. While the model suggests that firms developing CNS lead products were acquired for a − 29% discount relative to other disease areas, this was not significant (*p* = 0.153). Similarly, companies with orphan designated lead medicines were valued 18% higher, yet not significantly (*p* = 0.114). Overall, lead product characteristics explain 48.5% in value variation (Model 1).

#### Valuing further products

On average, company valuations increased by 15.2% (*p* < 0.001) for each additional product under development. In contrast, the average development score and the average number of indications do not seem to impact transaction value. Considering the high correlation of these insignificant variables with the lead product’s stage and indications, this result was expected. In conclusion, the total number of drugs improved adjusted-*R*^2^ by 4.9% (Model 3).

#### Transaction characteristics

Valuations were 63.4% (*p* < 0.001) higher for target companies located in the US relative to Europe, but not significantly higher for public targets (18.9%, *p* = 0.175). Furthermore, the acquirer’s market capitalization was identified as a major value driver, given that large cap corporations purchased Biopharma companies for a 73.2% (*p* < 0.001) premium compared to medium and small cap acquirers. The average market condition for acquisitions was favorable in the sample. Yet, better market conditions were considerably positively correlated with company valuations (*p* < 0.01). Lastly, spin-offs and single drug acquisitions were valued at a 40.4% (*p* < 0.05) discount relative to acquisitions of entire corporations. In summary, considering acquisition characteristics raised the adjusted-*R*^2^ by 11.1% (Model 4).

#### Further considerations

Model 5 excludes collinear variables with a Pearson correlation coefficient beyond 0.40 (average development score, average number of indications, and target ownership status). Thereby, the adjusted-*R*^2^ decreased by 0.1% to 64.4%. It is furthermore noteworthy that several independent variables display low, yet statistically significant, Pearson correlation coefficients (Supplementary Table e2). Transitions in-between development stages, e.g., Pre-Clinic to Phase 1 or Phases 2–3, differed significantly—solely the transition between Phase 3 to Approved was insignificant (*p* = 0.175).

A similar multivariate regression model for up-front payments can be found in the Supplementary Table e4. The overall model fit is similar, yet slightly higher than the total transaction value model (adjusted-*R*^2^ of 65.8%). Variable signs, magnitude, and significance levels follow the same concept explained for the total transaction value regression. Solely, the lead product’s number of indications and market conditions variables are slightly insignificant, while the spin-off variable turned significant.

## Discussion

Based on a sample of 311 Biopharma acquisitions from 2005 to 2020, mean valuations significantly rose for corporations with lead products in Pre-Clinic ($88 million), Phase 1 ($354 million), Phase 2 ($683 million), Phase 3 ($1761 million), and FDA Approved ($2469 million) development. Approximately, half of the agreed company valuation was deferred through regulatory and sales milestone payments for early development stages (Pre-Clinic and Phase 1). The lead drug’s molecule type and number of indications were positively correlated with company valuations. In addition, the total number of further products, targets headquartered in US, underlying market conditions, and acquirer market capitalization were estimated to have a significant positive impact on valuations.

These figures are in line with mean Biopharma acquisition valuations found in annually published M&A reports [10, 21]. The additional information gained about new drugs by conducting clinical trials is priced in by investors. In contrast, licensing agreements were more frequent than acquisitions in the examined period, yet their contract value—ranging from $20 million (Pre-Clinic) to $140 million (Phase 3)—was lower. Licensing agreements incur reduced valuations because contracts only incorporate distinct drug candidates, are subject to regional restrictions, and vary according to milestone thresholds and revenue distributions [28].

Similar to Rooswinkel et al. [24], we did not find a significant valuation difference between companies developing orphan and non-orphan lead products. Nonetheless, several factors positively affect the economics of orphan drugs: shorter development and approval timelines, additional financial R&D incentives, higher clinical trial and FDA success rates, stricter and extended market exclusivity, lower marketing costs, faster uptake, and high reimbursed prices [29–31]. Arguably, these factors could only significantly impact valuation in later development stages. Additionally, orphans’ market niche limits the number of strategic acquirers and restricts the eligible patient population. Combined with increasing pricing pressure [32], these factors could partially offset the favorably economics of orphan drugs.

In 2019, all top 10 grossing drugs were approved across several indications. Especially, treatments targeting molecular pathways that are inherent to multiple diseases, e.g., cancer or autoimmune diseases, may offer therapeutic benefits across several indications. Consequently, multi-indication drugs target an expanded patient group. However, early drug development timelines and costs of multi-indication drugs can be dynamically reduced as they only occur once per drug [33]. Additionally, the sequencing of indication launches permits higher pricing and revenues under single-price policies [34–36]. These financial factors offer explanations as to why Biopharma acquirers pay the observed 12% premium per additional indication for firms with multi-indication products.

Oncology, CNS, and anti-infective drugs were previously identified as key focus areas of large Biopharma companies with higher multiples [21, 26]. However, after adjusting for further covariates, the lead product’s disease area did not significantly impact company valuations. An orphan designation status and the number of indications might already account for the most important drug characteristics implicitly impacting its economic properties, such as price and target patient population. Additionally, the strategic fit between the acquirer’s and target’s product portfolio could impact acquisition values more than the underlying disease area.

Equivalent to Guo et al. [22], the sample demonstrates that valuation is positively associated with the company’s drug portfolio size. They ran a regression on 114 Biopharma IPOs (1991–2000) and identified that the number of total products and their patent protection are correlated to company valuations. Consequently, Biopharma valuation can be regarded as the sum of all products, each with its distinct clinical and economic characteristics, within a company’s R&D portfolio [12].

Results reveal higher valuations for companies with biologic or gene therapy lead products relative to small-molecules. Biologics and gene therapies often offer enhanced clinical safety and efficacy, higher clinical and FDA success rates, and target diseases previously considered untreatable [29, 37–39]. However, greater drug prices resulting from increased development and productions costs alongside impractical administration routes and reimbursement barriers hinder widespread commercialisation [40, 41]. Besides the enhanced therapeutic benefits, strategic acquirers are seemingly willing to pay a premium for the scientific technology inherent to biologics and gene therapeutics [9].

In line with previous research [25, 42], we found more and higher valued acquisitions of US companies relative to their European peers. Arguably, US Biopharma clusters in San Francisco, Cambridge (US), and San Diego provide start-ups with better access to human, technological, financial, and social capital to foster scientific innovation than their European counterparts in Zurich, Cambridge (UK), and Munich [25]. Moreover, stricter legal barriers for conducting laboratory research and clinical trials in European countries could ultimately impact Biopharma’s operating costs, and thereby company valuations [43].

Results of this study permit policy makers to design incentives for corporations to steer drug development into areas of interest. Neurodegenerative disorders cause a significant burden of disease in the US and Europe, yet drug development in this area is lagging. In our analysis, we also observed lower company valuations for CNS drug development companies. This “troublesome disconnect” between patients’ needs and lagging drug development may be overcome by providing higher research grants, regulatory submission support, and patent term expansions for CNS drugs—similar to regulations incentivizing orphan drug development [44]. Results also demonstrate that anti-biotic and anti-viral drugs are not valued significantly higher than their peers, even though recent policies aimed to incentivize drug development in this area [45]. Consequently, novel approaches beyond financial incentives that de-link drug prices from commercial success such as health impact bonds, pooled funds, or health impact funds, could be explored. The dataset also demonstrated that the valuation gap between companies with Phase 1 and 2 drugs is only marginal. Targeted financial and regulatory support programs may help to overcome this pharmaceutical “valley of death” [46]. Governments should also explore anti-cyclical industrial policies as results demonstrate that valuation and thereby available capital for drug development companies is scarce during economic downturns.

### Limitations

This study has several limitations. First, undisclosed information may impact results. Undisclosed acquisition valuations in the examined period may result in an over- or underestimation of company valuations. Especially, acquisitions of small pre-clinical biotechnology companies may not be released, which could overestimate valuations at this development stage. Additionally, unnamed pre-clinical drug candidates could overestimate the impact of the total number of products on firm valuation.

Second, the geographic scope of our analyses is limited to European and US Biopharma companies. Further studies investigating Biopharma company valuations in Asia, Africa, and South America are of interest. The therapeutic scope of the analyses is limited to companies developing NME for therapeutic use. Value drivers of medical technology, generic, and over-the-counter companies are subject to future research. The dataset is limited to a cross-section of Biopharma company valuations. Future panel studies should therefore examine the impact of time-varying variables on firm value.

Third, further variables are necessary to fully explain valuation of Biopharma companies. Even though the regression explains approximately 65% of the variation in company valuation, 35% remains unexplained. Variables distinctly describing each drug’s clinical benefit, anticipated competition, and population size are missing. A drug’s peak sales volume is a key, yet difficult to estimate, variable combining all named elements.

Fourth, company valuation is furthermore subject to negotiations between acquirers and targets/backers. Therefore, bargaining power, negotiation skills, soft skills, personal and inter-firm networks may influence company valuations in an up- or downward manner [47–49]. The applied valuation methodology—NPV, rNPV, real options, venture capital methods—could furthermore influence company valuations [11–13].

Fifth, some FDA-approved products have already been marketed for several years and could be close to patent expiry. Therefore, the observed marginal increase in company valuation might stem from difference in the lead product’s remaining exclusivity period.

## Conclusion

Greater transparency throughout the R&D process is necessary to unravel and optimize the timelines and costs associated with introducing new drugs to market. Internal and external drug development consume many financial and human resources, yet it is important for entrepreneurs, regulators, and payers to understand their exact magnitude and value drivers. This research revealed that Biopharma company valuation is significantly correlated with the lead product’s development stage, number of indications, treatment type, product portfolio size, headquarter location, acquirer market capitalization, and market conditions. Policy makers are encouraged to design targeted pricing and industrial policies that incentivize the development of novel drugs in areas with high unmet needs.

## Supplementary Information

Below is the link to the electronic supplementary material.

Supplementary file1 (PDF 513 kb)

## Data Availability

Not applicable.
