# Supplementary material for: Value drivers of development stage biopharma companies
Source: Eur J Health Econ. 2022 Jan 17;23(8):1287–96. doi: 10.1007/s10198-021-01427-5 (PMC9550717; doi:10.1007/s10198-021-01427-5)
Supplement: Supplementary file 1 — Supplementary file1 (PDF 513 kb) [file 10198_2021_1427_MOESM1_ESM.pdf]

Supplementary Online Content

Michaeli, D.M., Yagmur, H.B., Achmadeev, T., Michaeli, T. (2022). Value drivers of development stage biopharma companies. *The European Journal of Health Economics*. doi:10.1007/s10198-021-01427-5

Box e1 Mathematical equations for the regression analysis of acquisition value on collected variables

Table e1 Pearson correlation matrix for the sample of collected biopharma M&As

Table e2 Variance inflation factors for the regression of total transaction value on collected variables

Table e3 Omitted variable, model specification, heteroscedasticity, skewness, and kurtosis tests for the total transaction value regression

Table e4 Multivariate regression of up-front payment on lead product's, other products', and acquisition characteristics

This supplementary material has been provided by the authors to give readers additional information about their work.

The dependent variable (Y) was defined as company acquisition value (total acquisition value or up-front payment). Summary characteristics of the independent variables (x_i) are presented in Table 1. The association of independent variables with the dependent variable was examined in a sequence of regression models:

Model 1

Model 1 includes the lead product's development stage as the sole explanatory variables. Correlation coefficients are presented as α_k.

$$Log(Y = Company\ Valuation \mid X_i = x_i) = \alpha_0 + x_1\alpha_{Pre-Clinic} + x_1\alpha_{Phase\ 1} + x_2\alpha_{Phase\ 2} + x_3\alpha_{Phase\ 3} + x_4\alpha_{Approved}$$

Model 2

Model 2 includes all lead product characteristics as explanatory variables.

$$Log(Y = Company\ Valuation \mid X_i = x_i) = \alpha_0 + x_1\alpha_{Pre-Clinic} + x_1\alpha_{Phase\ 1} + x_2\alpha_{Phase\ 2} + x_3\alpha_{Phase\ 3} + x_4\alpha_{Approved} + x_5\alpha_{No.\ of\ Indications} + x_6\alpha_{Biologic/Gene\ Therapy} + x_7\alpha_{Oncology} + x_8\alpha_{CNS} + x_9\alpha_{Anti-Viral/Anti-Biotic} + x_{10}\alpha_{Orphan\ Designation}$$

Model 2

Model 3 includes all lead product and other product characteristics as explanatory variables.

$$Log(Y = Company\ Valuation \mid X_i = x_i) = \alpha_0 + x_1\alpha_{Pre-Clinic} + x_1\alpha_{Phase\ 1} + x_2\alpha_{Phase\ 2} + x_3\alpha_{Phase\ 3} + x_4\alpha_{Approved} + x_5\alpha_{No.\ of\ Indications} + x_6\alpha_{Biologic/Gene\ Therapy} + x_7\alpha_{Oncology} + x_8\alpha_{CNS} + x_9\alpha_{Anti-Viral/Anti-Biotic} + x_{10}\alpha_{Orphan\ Designation} + x_{11}ß_{Total\ No.\ of\ Drugs} + x_{12}ß_{Average\ Development\ Score} + x_{13}ß_{Average\ No.\ of\ Indications}$$

Model 4

Model 4 includes all lead product, other product, and acquisition characteristics as explanatory variables.

$$Log(Y = Company\ Valuation \mid X_i = x_i) = \alpha_0 + x_1\alpha_{Pre-Clinic} + x_1\alpha_{Phase\ 1} + x_2\alpha_{Phase\ 2} + x_3\alpha_{Phase\ 3} + x_4\alpha_{Approved} + x_5\alpha_{No.\ of\ Indications} + x_6\alpha_{Biologic/Gene\ Therapy} + x_7\alpha_{Oncology} + x_8\alpha_{CNS} + x_9\alpha_{Anti-Viral/Anti-Biotic} + x_{10}\alpha_{Orphan\ Designation} + x_{11}ß_{Total\ No.\ of\ Drugs} + x_{12}ß_{Average\ Development\ Score} + x_{13}ß_{Average\ No.\ of\ Indications} + x_{14}\gamma_{Target\ HQ\ in\ US} + x_{15}\gamma_{Target\ Public\ Ownership} + x_{16}\gamma_{Acquirer\ Market\ Cap\ \geq \$10\ Bn} + x_{17}\gamma_{Market\ Conditions} + x_{18}\gamma_{Spin-Off/Single\ Drug\ Deal}$$

Model 5

Model 5 includes for collinearity selected lead product, other product, and acquisition characteristics as explanatory variables.

$$Log(Y = Company\ Valuation \mid X_i = x_i) = \alpha_0 + x_1\alpha_{Pre-Clinic} + x_1\alpha_{Phase\ 1} + x_2\alpha_{Phase\ 2} + x_3\alpha_{Phase\ 3} + x_4\alpha_{Approved} + x_5\alpha_{No.\ of\ Indications} + x_6\alpha_{Biologic/Gene\ Therapy} + x_7\alpha_{Oncology} + x_8\alpha_{CNS} + x_9\alpha_{Anti-Viral/Anti-Biotic} + x_{10}\alpha_{Orphan\ Designation} + x_{11}ß_{Total\ No.\ of\ Drugs} + x_{14}\gamma_{Target\ HQ\ in\ US} + x_{16}\gamma_{Acquirer\ Market\ Cap\ \geq \$10\ Bn} + x_{17}\gamma_{Market\ Conditions} + x_{18}\gamma_{Spin-Off/Single\ Drug\ Deal}$$

Regression coefficients are presented as α_k for lead product, $ß_k$ for other product, and γ_k for acquisition characteristics.

Box e1 Mathematical equations for the regression analysis of acquisition value on collected variables

CNS central nervous system, *FDA* US Food and Drug Administration, *HQ* headquarter.

	(1)	(2)	(3)	(4)	(5)	(6)	(7)	(8)	(9)	(10)	(11)	(12)	(13)	(14)	(15)	(16)	(17)
(1) Log(total transaction value)	1																
(2) Log(up-front payment)	0.89***	1															
(3) Lead development phase	0.66***	0.68***	1														
(4) Lead no. of indications	0.31***	0.29***	0.25***	1													
(5) Biologic/gene therapy	0.10	0.14*	-0.04	0.08	1												
(6) Oncology	0.03	0.02	-0.12*	0.19***	0.14*	1											
(7) CNS	-0.1	-0.09	0.00	-0.11*	-0.06	-0.29***	1										
(8) Anti-viral/anti-biotic	-0.05	-0.02	-0.07	-0.08	-0.09	-0.23***	-0.16**	1									
(9) FDA orphan designation	0.25***	0.23***	0.26***	0.24***	0.05	0.09	-0.08	-0.13*	1								
(10) Total no. of drugs	0.34***	0.39***	0.13*	0.09	0.02	0.06	-0.01	-0.05	0.11*	1							
(11) Mean development score [a]	0.44***	0.44***	0.83***	0.12*	-0.03	-0.15**	0.04	-0.06	0.19***	-0.26***	1						
(12) Mean no. of indications [b]	0.24***	0.20***	0.22***	0.82***	0.16**	0.13*	-0.1	-0.06	0.23***	-0.11*	0.24***	1					
(13) Target HQ US	0.22***	0.15*	0.10	-0.06	0.00	0.06	0.01	0.06	0.01	-0.13*	0.14*	-0.01	1				
(14) Target public	0.39***	0.46***	0.40***	0.15**	0.05	0.00	-0.01	0.00	0.08	0.29***	0.24***	0.06	0.15**	1			
(15) Acquirer market cap ≥ $10 Bn	0.44***	0.43***	0.22***	0.05	0.06	-0.06	0.00	-0.05	0.02	0.17**	0.1	-0.06	0.10	0.12*	1		
(16) Market condition	0.20***	0.19*	0.08	0.07	0.03	-0.07	-0.02	0.09	0.02	0.03	0.09	0.06	0.06	0.00	0.07	1	
(17) Spin-off/single drug deal	-0.09	-0.08	0.10	0.00	0.05	-0.05	-0.04	0.07	0.01	-0.13*	0.21***	0.11*	-0.04	0.05	-0.02	-0.07	1

Table e1 Pearson correlation matrix for the sample of collected biopharma acquisitions

CNS central nervous system, *FDA* US Food and Drug Administration, *HQ* headquarter.

[a] The average development score represents the number of years required to reach each development stage.

[b] The average number of indications refers to all products excluding the lead product.

	Model 1	Model 2	Model 3	Model 4	Model 5
Dependent variable: natural logarithm of total transaction value					
(**A**) Lead product					
Phase 1	1.61	1.66	1.98	2.05	1.72
Phase 2	2.15	2.24	4.07	4.28	2.39
Phase 3	1.81	1.92	5.24	5.64	2.04
Approved	1.89	2.14	11.35	12.04	2.38
No. of indications		1.17	3.79	3.89	1.20
Biologic/gene therapy		1.96	1.08	1.10	1.07
Oncology		1.35	1.35	1.40	1.40
CNS		1.20	1.21	1.22	1.21
Anti-viral/anti-biotic		1.17	1.17	1.20	1.20
FDA orphan designation		1.13	1.15	1.16	1.15
(**B**) Other products					
Total no. of drugs			2.02	2.13	1.12
Average development score [a]			7.36	7.56	
Average no. of indications [b]			3.64	3.73	
(**C**) Acquisition characteristics					
Target HQ US				1.13	1.09
Target public ownership				1.44	
Acquirer market cap ≥ $10 Bn				1.17	1.14
Market conditions				1.06	1.05
Spin-off/single drug acquisition				1.12	1.09
**Mean VIF**	**1.87**	**1.50**	**3.49**	**2.96**	**1.42**

Table e2 Variance inflation factors for the regression of total transaction value on collected variables

The Table presents VIF for each model of the conducted regression. VIF beyond 10 indicated multicollinearity between independent variables. *CNS* central nervous system, *FDA* US Food and Drug Administration, *HQ* headquarter, *VIF* variance inflation factors.

[a] The average development score represents the number of years required to reach each development stage.

[b] The average number of indications refers to all products excluding the lead product.

	**Model 1**	**Model 2**	**Model 3**	**Model 4**	**Model 5**
Ramsey's-test					
p value	NA	0.0468	0.3188	0.6531	0.7769
F value	NA	2.69	1.18	0.54	0.37
Link-test					
hat	0.122	0.003	0.001	0.005	0.001
hat²	1.000	0.391	0.405	0.481	0.929
Cameron & Trivedi's-test					
Heteroscedasticity	0.8356	0.4851	0.0747	0.3070	0.3400
Skewness	0.8445	0.1954	0. 894	0.7163	0.5276
Kurtosis	0.9589	0.7828	0.5081	0.1362	0.1914
**Total**	**0.9699**	**0.3917**	**0.0391**	**0.3640**	**0.3566**

Table e3 Omitted variable, model specification, heteroscedasticity, skewness, and kurtosis tests for the total acquisition value analysis

The Ramsey's-test was performed to detect omitted variables in the regression model (H_0: model has no omitted variables). The Link-test was conducted to identify model specification errors (H_0 of hat²: model has no specification errors). Cameron & Trivedi's-test was executed to identify heteroscedasticity, skewness, and kurtosis in our model (H_0: model has no heteroscedasticity, skewness, or kurtosis).

	Model 1	Model 2	Model 3	Model 4	Model 5
Dependent variable: natural logarithm of up-front payment					
(**A**) Lead product					
Phase 1	1.504***	1.482***	1.328***	0.871**	0.993***
Phase 2	2.144***	2.218***	1.976***	1.339***	1.618***
Phase 3	3.251***	3.324***	3.170***	2.138***	2.723***
Approved	4.108***	3.977***	3.711***	2.376***	3.293***
No. of indications		0.0994*	0.090*	0.047	0.0930*
Biologic/gene therapy		0.687***	0.678***	0.555***	0.603***
Oncology		0.217	0.140	0.184	0.181
CNS		-0.277	-0.295	-0.286	-0.257
Anti-viral/anti-biotic		0.341	0.357	0.363	0.376
FDA orphan designation		0.153	0.061	0.143	0.130
(**B**) Other products					
Total no. of drugs			0.251***	0.236***	0.223***
Average development score [a]				0.065	
Average no. of indications [b]				0.105	
(**C**) Acquisition characteristics					
Target HQ US				0.251	0.364*
Target public ownership				0.537**	
Acquirer market cap ≥ $10 Bn				0.901***	0.866***
Market conditions				0.625	0.599
Spin-off/single drug acquisition				-0.691**	-0.571*
Constant	2.938***	2.517***	2.018***	1.500***	1.728***
No. of observations	301	301	301	301	301
R^2	46.7%	51.7%	60.0%	69.2%	67.5%
Adjusted-R^2	45.9%	50.0%	58.4%	67.2%	65.8%
F test					
Pre-Clinic to Phase 1	1.504***	1.482***	1.263***	0.871**	0.993***
Phase 1 to 2	0.640**	0.736**	0.597**	0.468*	0.625**
Phase 2 to 3	1.106***	1.106***	1.116***	0.799***	1.105***
Phase 3 to Approved	0.857***	0.653**	0.429	0.238	0.570**

Table e4 Multivariate regression of up-front payment on (**A**) lead product's, (**B**) other products', and (**C**) acquisition characteristics

CNS: central nervous system; FDA: US Food and Drug Administration; HQ: headquarter. *p* values: * $p<0.05$, ** $p<0.01$, *** $p<0.001$, *NS* not significant.
[a] The average development score represents the number of years required to reach each development stage.
[b] The average number of indications refers to all products excluding the lead product.
